# Reassessment of different criteria for diagnosing post-hepatectomy liver failure: a single-center study of 1683 hepatectomy

**DOI:** 10.18632/oncotarget.19360

**Published:** 2017-07-18

**Authors:** Yongchang Zheng, Huayu Yang, Li He, Yilei Mao, Hanze Zhang, Haitao Zhao, Shunda Du, Yiyao Xu, Tianyi Chi, Haifeng Xu, Xin Lu, Xinting Sang, Shouxian Zhong

**Affiliations:** ^1^ Department of Liver Surgery, Peking Union Medical College Hospital, Chinese Academy of Medical Sciences and Peking Union Medical College (CAMS & PUMC), Beijing, China; ^2^ Department of Ophthalmology, School of Medicine, Emory University, Atlanta, Georgia, USA; ^3^ Department of Epidemiology and Biostatistics, College of Public Health, University of South Florida, Tampa, Florida, USA

**Keywords:** PHLF, ISGLS, child-pugh score, “50-50” criteria, Clavien-Dindo

## Abstract

Assessing the incidence and severity of post-hepatectomy liver failure (PHLF) can be based on different criteria, and we wished to compare the diagnostic efficiency and specificity of different PHLF criteria. Data from patients (*n*=1683) who received hepatectomies in the liver surgery department of Peking Union Medical College Hospital from April 2008 to August 2014 were retrospectively analyzed. Possible PHLF patients were screened according to the criteria of the International Study Group of Liver Surgery (ISGLS). Subsequently, other PHLF evaluation methods, including Child-Pugh score, “50-50” criteria, Model for End-Stage Liver Disease (MELD) score, and Clavien-Dindo classification were used to assess the suspected PHLF patients, and statistical analysis was performed for correlation of these methods with clinical prognoses. Using ISGLS grading, 40 cases (2.38%) were suspected to have PHLF, among whom 5 (0.30%) patients died. Of the 40 cases there were 9 patients of ISGLS grade A, 21 of grade B, and 10 of grade C. Among the entire group, Child-Pugh scoring showed 3 patients in grade A, 35 in grade B, and 2 in grade C, while only 5 patients met the “50-50” criteria. Interestingly, MELD scores ≥11 points were found only in 3 cases. Twenty-eight patients were classified as Clavien-Dindo grade I, 8 as grade II, 3 as grade III, and 1 as grade IV. Prothrombin time on postoperative day 5 (PT5), ISGLS, and Clavien-Dindo were found to have significant correlation with the prognosis of PHLF (*r*>0.5, *p* <0.05), thus can be used as prognosis predictors for PHLF patients.

## INTRODUCTION

Hepatocellular carcinoma (HCC) is the third leading cause of death from cancer worldwide [1, 2]. In the United States, HCC has steadily increased in annual incidence, and it is anticipated to become more prevalent in the next 20 years due to the increasing onset of steatohepatitis and diabetes [3, 4]. Surgical resection remains one of the most effective treatments for hepatic tumors among various treatment options [5]. The advent of innovations in surgical technique and specialized medical devices has made hepatectomy a relatively safe procedure. However, mortality after hepatectomy still occurs due to various factors, the most significant of which is insufficient postoperative liver function, or so called post-hepatectomy liver failure (PHLF) [6]. Methods that are currently used in clinical practice for PHLF diagnosis or liver function evaluation include the Child-Pugh score [7], “50-50” criteria [8], MELD (Model For End-Stage Liver Disease) score [9], and Clavien-Dindo classification [10]. Each method was developed from a single institute or hospital with a limited number of patients, which has led to substantial differences in the reported incidence of PHLF cases (1.2-32%) [11-17]. The International Study Group of Liver Surgery (ISGLS) developed a new method for PHLF grading in 2011 [18] with the collective effort of collaborators from 10 countries. However, there is still a lack of unified definition for PHLF (e.g., varying standards for time point and severity) and acceptable quantitative criteria (such as lab test values and other clinical observation indices) worldwide. Meanwhile, with the advances in surgical technique, preoperative assessment, and postoperative liver function protection in recent years, there is a critical need to reassess the actual incidence of liver failure following hepatectomy. The purpose of the present study is to assess the current incidence and severity of PHLF with the recently-developed ISGLS methods, to compare different PHLF Criteria, and to raise useful suggestions for the diagnosis and treatment of PHLF.

Because of the infrequent morbidity with the development of new techniques in liver surgery, as well as the difficulty in predicting the occurrence of PHLF before surgery, this study could not be designed as a prospective clinical trial. Therefore, the evidence could only be accumulated from large case summaries. We retrospectively reviewed 1683 cases of hepatectomy that were performed in the Peking Union Medical College Hospital from April 2008 to August 2014. Possible PHLF patients were screened in reference to the ISGLS guidelines. Other methods, including the Child-Pugh score, “50-50” criteria, MELD score, and the Clavien-Dindo classification were assessed for their accuracy and validity.

## MATERIALS AND METHODS

We retrospectively reviewed all hepatectomy surgeries performed in our department from April 2008 to August 2014. The demographic data for the 1683 patients are listed in Table 1. Among the 1683 cases of hepatectomy, final pathological diagnosis proved hepatocellular carcinoma in 581 cases, adenocarcinoma in 142 cases, cholangiocarcinoma in 93 cases, cavernous hemangioma in 374 cases, and other tumors (such as neuroendocrine tumors) in 493 cases. Types of surgery included hemihepatectomy in 207 cases, multiple segment resection in 767 cases, single segment resection in 187 cases, and simple removal of tumor in 522 cases (Table 1). Cirrhosis was found in 342 cases (including 295 cases from hepatitis B, 28 cases from hepatitis C, and 19 cases from other causes). In all the patients, liver function including lab tests and remnant liver volume were carefully assessed and estimated. Most of the patients had Child-Pugh grade A liver function and remnant liver function was enough for patient survival based on current criteria or expert experience.

**Table 1:** Demographic information of 1683 patients and their related surgical pathology

Variables	Value
Age: median (range)	53 (12-86)
Gender	
Male	900 (53.48%)
Female	783 (46.52%)
**Type of Surgery:**	
Simple removal of tumor	522 (31.02%)
Single segment resection	187 (11.11%)
Multiple segment resection	767 (45.57%)
Hemihepatectomy	207 (12.30%)
**Liver lesions:**	
HCC	581 (34.52%)
Cavernous hemangioma	374 (22.22%)
Cholangiocarcinoma	93 (5.53%)
Adenoma	142 (8.44%)
Other tumors	493 (29.29%)

Numbers were shown as Median (range). HCC: Hepatocellular carcinoma.

All patients received routine postsurgical treatment during the early recovery phase. Early enteral nutrition was administered immediately after gut motility was restored, and albumin was given to patients with low serum albumin levels. All postoperative statuses and treatments were also recorded and evaluated. Serological tests for serum total bilirubin (TBil), prothrombin time (PT), and blood coagulation were performed during the postoperative recovery period.

All 1683 patients were analyzed and screened with reference to ISGLS criteria for PHLF. The patients’ data were collected from the perioperative phase and the postoperative phase and were used to assess the effectiveness and validity of other methods including Child-Pugh, “50-50” criteria, MELD score, and Clavien-Dindo. Statistical analysis was performed for the incidence of PHLF, its related risk factors, and prognostic factors.

Values are presented as mean ± standard deviation or median (range) median ± Interquartile range (IQR). Group differences in deceased PHLF patients and PHLF survivors were tested using the Mann-Whitney U test. Spearman correlation coefficients were used to analyze relationships between the lab test values, various PHLF criteria of postoperative day 5, and clinical outcome. The receiver operating characteristic (ROC) curves were constructed to evaluate the PHLF predictive values of PT, ISGLS, Clavien-Dindo, Child-Pugh, and MELD. A two-tailed *p* value of < 0.05 was considered to be of statistical significance. All analyses were performed using SPSS 12.0 (SPSS, Cary, North Carolina, USA).

## RESULTS

According to ISGLS criteria, 40 patients were identified as suspected PHLF cases through screening, among which 5 (12.50%) patients died of PHLF. The suspected PHLF cases accounted for 2.38% (40 of 1683) of all hepatectomy patients. Total post-hepatectomy mortality due to all causes was 0.77% (13 of 1683), compared to a post-hepatectomy mortality of 0.30% (5 of 1683) due to PHLF. Preoperational data for the 40 suspected PHLF patients are shown in Table 2. Among the 40 suspected PHLF patients, 25 (62.5%) had hepatitis B, 7 (17.5%) had hepatitis C, and 8 (20.00%) were negative for both hepatitis viruses. Three patients (7.50%) were treated with preoperative radiofrequency ablation while 4 (10.00%) received another preoperative intervention. Preoperative imaging showed that 5 (12.50%) patients had vascular tumor thrombus and 4 (10.00%) patients had tumor thrombus in the bile duct. The mean TBil before surgery was 31.74 μmol/L, and average PT was 13.08 s (Table 2).

**Table 2:** Demographic information and preoperational data of PHLF patients.

Variables	40 PHLF patients		
Age: median (range)	59.5 (39-74)		
Gender			
Male	32 (80.00%)		
Female	8 (20.00%)		
Hepatitis B (HbsAg+)	25 (62.50%)		
HBV-DNA<1000IU/ml	14 (35.00%)		
HBV-DNA>1000IU/ml	11 (27.50%)		
Liver functional status			
Child-Pugh class A	26 (65.00%)		
Child-Pugh class B	14 (35.00%)		
Liver function tests			
ALT (U/L)	72.25 (10-667)		
TBil (μmol/L)	31.74 (7.7-154.6)		
ALB (g/L)	37.18 (25-46)		
PT (s)	13.08 (10.5-17.3)		
Cirrhosis	25 (62.50%)		
Tumor nodules			
N=1	30 (75.00%)		
N≥2	10 (25.00%)		
Operation time (min)	295.5 (90-575)		
**Type of Surgery**	**PHLF cases**	**Total cases**	**Incidence of PHLF**
Simple removal of tumor	9	522	1.72%
Single segment resection	5	187	2.67%
Multiple segment resection	13	767	1.69%
Hemihepatectomy	13	207	6,28%
Other parameters			
Pringle's maneuver	21(52.50%)		
Ischemic duration (min)	24.90 (11-57)		
Blood loss (ml)	1110.00 (100-8000)		
Patients transfused (n)	30(75.00%)		
RBC (unit)	6.23 (2-30)		
Serum (ml)	865.47 (300-2800)		

Numbers were shown as median (range). HBV: Hepatitis B Virus, RBC: Red Blood Cell.

The biochemical tests performed on postoperative day 5 are listed in Table 3. By statistical analysis, no significant differences were found except in PT and PT% between deceased and surviving patients (Table 3). Pathology results of the 40 PHLF patients showed 31 (77.50%) with HCC, 3 (7.50%) with cholangiocarcinoma, 1 (2.50%) with hepatitis, 1 (2.50%) with biliary mucosa inflammation, 2 (5.00%) with cavernous hemangioma, 1 (2.50%) with metastatic cancer, and 1 (2.50%) with hepatic hydatid disease.

**Table 3:** Biochemistry test results on day 5 after surgery of the 40 PHLF patients.

Tests	5 Deceased patients	35 PHLF survivors	*p* value
TBil (μmol/L)	86.0 (50.2-347)	67.8 (50.8-215.6)	0.363
PT (s)	18.1 (16.6-30.7)	14.8 (12.2-18.3)	0.013
PT (%)	46.4 (27.1-56.3)	63.3 (46.1-85.6)	0.004
ALB (g/L)	35 .0 (25-40)	35 .0 (27-41)	0.875
Cr (μmol/L)	64 .0 (58-87)	61 .0 (41-146)	0.346

Numbers were shown as Median (range). Tbil: Total bilirubin, PT: Prothrombin time, Alb: Albumin, Cr: Creatinine.

The 40 suspected PHLF patients were evaluated with various criteria and clinical data on postoperative day 5. The data are listed separately for the 5 deceased and 35 surviving patients. All 40 patients were found to fit into one of the three Child-Pugh score categories. However, only 5 patients met the “50-50” criteria, and 3 of those died of PHLF. The MELD score was set with a cut-off line of 11 and showed that 37 (92.50%) patients had scores that were lower than 11. Four of the 5 deceased patients had a MELD score lower than 11. With the Clavien-Dindo criteria, 3 of the deceased patients fell into grade II (60%), 1 into grade IIIa (20%), and 1 into grade IVa (20%) (Table 4).

**Table 4:** Assessment of the 40 suspected PHLF patients with currently-used clinical criteria.

Variables	5 deceased patients	35 PHLF survivors
Child-Pugh Score		
Child-Pugh grade A	0	3 (8.57%)
Child-Pugh grade B	3 (60.00%)	32 (91.43%)
Child-Pugh grade C	2 (40.00%)	0
“50-50” Score		
PT>50+TBil>50	3 (60.00%)	2 (5.71%)
ISGLS criteria		
A	0	9 (25.71%)
B	0	21 (60.00%)
C	5 (100.00%)	5 (14.29%)
MELD Score		
MELD<11	4 (80.00%)	33 (94.29%)
MELD≥11	1 (20.00%)	2 (5.71%)
Clavien-Dindo Classification		
I	0	28 (80.00%)
II	3 (60.00%)	5 (14.29%)
IIIa	1 (20.00%)	0
IIIb	0	2 (5.71%)
IVa	1(20.00%)	0
IVb	0	0
V	0	0

ISGLS: International Study Group of Liver Surgery, MELD: Model for End-stage Liver Disease.

We performed univariate correlation analysis for the laboratory tests (serum bilirubin, albumin, coagulation) on postoperative day 5 as well as for the currently-used clinical liver function assessment methods including Child-Pugh score, ISGLS criteria, MELD score, “50-50” score, and Clavien-Dindo classification with the prognosis of the suspected PHLF patients. We found that PT5 level, Child-Pugh score, ISGLS criteria, MELD score, and Clavien-Dindo classification had a statistically significant correlation with the final outcome of the patients, with the PT5 level, ISGLS criteria, and Clavien-Dindo classification having the highest correlation coefficients (r > 0.5) (Table 5).

**Table 5:** Univariate analysis of correlation of each liver function indicator and criteria with clinical outcomes

Indicators	PT5	Child-Pugh	ISGLS	MELD	Clavien-Dindo
**Correlation index**	0.642	0.434	0.535	0.409	0.602
***P***	0.000	0.005	0.000	0.009	0.000

PT5: Prothrombin Time on postoperative day 5, ISGLS: International Study Group of Liver Surgery, MELD: Model for End-stage Liver Disease.

**Figure 1:**
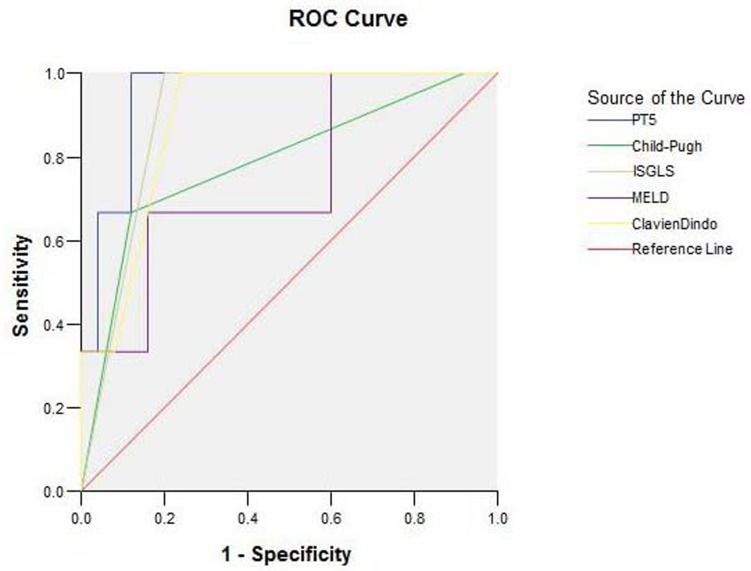
ROC curve of PT5, ISGLS, Clavien-Dindo, Child-Pugh, and MELD The area under curve (AUC) for PT5, ISGLS, Clavien-Dindo, Child-Pugh, and MELD were 0.947±0.046 (95%CI: 0.857-1.037; *p* = 0.013), 0.900±0.061 (95%CI: 0.780-1.020; *p* = 0.026), 0.893±0.069 (95%CI: 0.757-1.029; *p* = 0.028), 0.787±0.156 (95%CI: 0.481-1.092; *p* = 0.110), and 0.747±0.157 (95%CI: 0.439-1.054; *p* = 0.169), respectively. The PT5 level exhibited the largest AUC value and highest prediction power, followed by ISGLS, Clavien-Dindo, Child-Pugh, and MELD. PT5: PT on postoperative day 5.

We further analyzed these five indicators as predictors for clinical outcomes using receiver operating characteristic (ROC) curves, and the results were shown in Figure 1 (50-50 criteria not included due to very limited case number). The area under curve (AUC) for PT5, ISGLS, Clavien-Dindo, Child-Pugh, and MELD were 0.947±0.046 (95%CI: 0.857-1.037; *p* = 0.013), 0.900±0.061 (95%CI: 0.780-1.020; *p* = 0.026), 0.893±0.069 (95%CI: 0.757-1.029; *p* = 0.028), 0.787±0.156 (95%CI: 0.481-1.092; *p* = 0.110), and 0.747±0.157 (95%CI: 0.439-1.054; *p* = 0.169), respectively. Overall, the PT5 level exhibited the largest AUC value and highest prediction power, followed by ISGLS, Clavien-Dindo, Child-Pugh, then MELD. Specifically, the sensitivity of PT5 testing for PHLF was 66.7% and specificity was 96.0% with a cut-off value of 17.15. When the sensitivity was 33.3%, specificity became 100% with a cut-off value of 24.05 (Figure 1).

## DISCUSSION

PHLF has been a major concern and cause of hepatectomy-related death over the past few decades [11, 14, 19, 20]. Different incidence and mortality rates have been reported based on different liver remnant volumes and patients’ respective conditions [21-23]. However, there are no universally standardized definitions and diagnosis criteria for PHLF.

In this study, we screened 1683 hepatectomy patients with reference to ISGLS criteria and only 40 patients were in line with the criteria. The incidence of PHLF in our study (2.38%) is lower than most of published rates (1.2-32%) [11-17]. There were no significant differences between these 40 patients and the other surgical patients in their baseline status and in some surgical conditions including surgical duration, ischemic time, and blood loss.

Considering that resection of large portions of liver may cause loss of normal liver tissue, increased hemorrhage, and large operational wound, etc., the extent of liver resection is considered an important factor which correlates with the post-operational outcome and onset of PHLF [11, 19]. Consistent results were also found in our study: hemihepatectomy with the largest resection extent led to the highest PHLF incidence (6.28%), which was significantly higher than single tumor removal (1.72%, adjusted *p* = 0.0036, chi-square test) and multiple segment resection (1.69%, adjusted *p* = 0.0008). Compared to single segment resection (2.67%), hemihepatectomy also caused higher (but statistically insignificant) incidence of PHLF (adjusted *p* = 0.26, which may be caused by a relatively small sample size). However, we also admit the extent of liver resection mainly depends on the primary disease and surgical indication, which should have a strong correlation with PHLF. For example, PHLF risk of a hemihepatectomy on haemangioma of a normal liver would be totally different from that of a hemihepatectomy on HCC combined with cirrhosis. Therefore, the extent of liver resection may be a primary-disease-dependent correlation factor for the onset of PHLF.

Among all patients, the total postsurgical mortality was 13, where 8 patients died of causes other than PHLF (pulmonary infection: 3; acute myocardial infarction: 1; bleeding: 2; pulmonary embolism: 2). Only 5 patients died of PHLF, accounting for only 0.30% of overall post-hepatectomy cases. This was much lower than the previous reported mortality rate (3.40%) attributable to PHLF [8]. Many PHLF cases tended to be “atypical.” The vast majority of the suspected PHLF patients (87.50%) in our study have completely recovered. In fact, these patients’ cases might not be qualified as PHLF if the definition were set differently. After nearly 10 years of developments in preoperative evaluation of liver surgery [24, 25], surgical procedures, and perioperative management, the incidence of PHLF has decreased dramatically, at least in major liver surgery centers. Therefore, the clinical significance of PHLF may have already changed. Our study provides evidence that PHLF could be recognized as a relatively rare complication of hepatectomy.

The Child-Pugh score may be a more useful method in assessing the risks and applicability of surgery preoperatively rather than postoperatively. In this study, all patients who were preoperatively assessed as Child-Pugh grade A recovered well and were discharged from the hospital. The deceased patients obtained Child-Pugh grade B or above in both preoperative and postoperative evaluation. Due to the relatively loose criteria of Child-Pugh scoring, very few patients were classified into a higher grade (grade C) (2 of 40, 5.00%). Though it is a traditional and widely-used liver function evaluation method, the Child-Pugh score correlated less strongly with postoperative clinical outcomes compared to other, newly-developed classification criteria (Table 5).

The “50-50” standard was established based on the data from a single study of 775 patients between 1998-2002, and was claimed as an accurate early indicator of PHLF and predictor of 50% mortality [8]. Another validation study involving 807 patients reported that the 50-50 method may be less meaningful as a predictive marker for PHLF but may reflect the risk of mortality [26]. In our study, only 5 out of the 40 PHLF patients met the “50-50” criteria, indicating its low sensitivity for predicting the morbidity of PHLF. However, 3 (60.00%) of the 5 patients died, suggesting its possible correlation with mortality of PHLF, which tends to agree with conclusions from some previous studies. No statistical conclusion can be drawn due to the limited case number in our study. It is worth mentioning that, a few advantages of 50-50 criteria is its postoperative time frame (postoperative day 5) and quantitative values (PT < 50% and SB > 50 μml/L) for determination of PHLF, which happen to be absent in other assessment criteria including ISGLS and Clavien-Dindo. Considering the inconsistent findings using 50-50 criteria in different series of cases, further validation is warranted from future studies at multiple institutes.

MELD score has been the most commonly used method for liver function evaluation in liver transplantation. However, it seems less effective as a factor for prognosis of PHLF since 80% of the deceased patients had a score lower than 11. The creatinine value (Cr) is a weighted factor in the MELD score system, but the elevation of Cr is uncommon in the early stages of PHLF. Our data also indicate that Cr value has low correlation with clinical outcomes, and that there is no difference in Cr values between deceased PHLF patients and survivors.

We used ISGLS criteria for patient screening, both for its relatively wide acceptance and more recent development. Despite these advantages of ISGLS criteria, the lack of quantitative indices for assessment may still be a source of confusion in clinical practice. Of the 5 patients that died of PHLF, all fell into grade C of ISGLS criteria, accounting for 50% of surviving and non-surviving grade C patients. There seems to be an association between PHLF patients fulfilling grade C of ISGLS criteria and higher mortality. Nonetheless, we cannot make a definite conclusion due to the small number of cases. Meanwhile, we must admit that PHLF patients fulfilling grade C of ISGLS criteria would have had very severe multi-organ dysfunction and/or failure and require intensive care; thus, usage of ISGLS grade C as a predictor of the PHLF outcome should be further evaluated with more cases.

It was very interesting to find that the Clavien-Dindo classification also had good correlation with clinical outcomes in our study. However, Clavien-Dindo classification is a common classification system for evaluating the severity of operative trauma and is not specific for liver surgery. The results shown here indicate that the severity of Clavien-Dindo score may be related to postoperative liver function as well as PHLF.

The PT is mainly controlled by the liver’s synthesis of coagulation factors II, VII, I X and X; thus, PT is an important indicator of liver function. One important finding in our study is that the clinical outcomes of PHLF patients were significantly correlated with the values of the PT level on postoperative day 5. ROC analysis also suggested that PT5 was significantly associated with the outcome of PHLF. Because it is an easily-performed test, PT5 could be a single valuable indicator for PHLF, which may be useful for the identification and monitoring of potential PHLF patients. In detail, our study showed that the mean PT5 value obtained from PHLF patients was higher compared to the mean PT value obtained before surgery. The PT5 value of non-PHLF patients was 12.06, which was significantly lower than that of PHLF patients (18.1 in 5 deceased PHLF patients and 14.8 in 35 PHLF survivors).

Bilirubin is another commonly-used serological marker for PHLF assessment. Previous reports have set bilirubin concentrations from 2 mg/dl [27] up to 14 mg/dl [28] as a threshold for PHLF, while most literature has set 50 μmol/L (2.92 mg/dl) as a threshold for PHLF [18, 29]. Based on our observations, the level of total bilirubin increased 5-10 days after hepatectomy in all 40 patients. The average level of blood total bilirubin was 67.8 μmol/L in the 35 surviving PHLF patients and 86.0 μmol/L in the 5 deceased patients on postoperative day 5. It is noticeable that a further elevated total bilirubin level existed in all 5 deceased patients than in the 35 surviving PHLF patients, although no statistically significant difference was found due to the limited number of patients. We presume a constantly elevated level of blood total bilirubin may indicate poor prognosis of PHLF patients, but more robust evidence from large study series is needed.

From our observations and series analysis, each method we evaluated in this study has certain limitations. We would like to point out that, the Child-Pugh score may be a combination of many clinical parameters, but it failed to correlate significantly with outcome. The Child-Pugh score is based on the quantitative results of serum bilirubin and PT levels. These individual markers, when used appropriately, could be more accurate and faster for PHLF assessment. As suggested by our study, the constant rising of total bilirubin and prolonged PT on postoperative day 5 may be valuable tools for predicating the onset and outcome of PHLF.

Due to the small number of cases in our study, further clinical research is still needed, especially across multiple medical centers. The limited available data might be part of the reason for the inability to determine a satisfactory and broadly applicable evaluation method for PHLF. From the perspective of our study, the incidence of PHLF is fairly low in major medical centers; thus sampling size in future PHLF studies may be correspondingly low, causing a dilemma for the study of PHLF.

In summary, certain limitations exist in each of the currently available evaluation methods for PHLF. From our hepatectomy series, we propose that the incidence of PHLF has decreased greatly in recent years, and a much lower mortality rate was observed in our study. Great efforts should be focused on preoperative and intraoperative assessment for the possibility of PHLF. Furthermore, we suggest that PT level on postoperative day 5 may be crucial for the diagnosis and outcome prediction of PHLF, and those other liver function markers such as serum total bilirubin should also be taken into consideration.
